# Evaluating the effect of village health workers on hospital admission rates and their economic impact in the Kingdom of Bhutan

**DOI:** 10.1186/s12889-020-09347-4

**Published:** 2020-08-24

**Authors:** Sacha C. Hauc, Dolley Tshering, Josemari Feliciano, Agata M. P. Atayde, Layla M. Aboukhater, Kinley Dorjee, Tshering Dukpa, Pema Rinchen, Neema Yoezer, Casey M. Luc, Rup N. Adhikari, Kezang Lhamo, Kaveh Khoshnood

**Affiliations:** 1 Yale University School of Public Health, Laboratory of Epidemiology and Public Health, 60 College St, New Haven, CT 06510 USA; 2 Bhutan Ministry of Health, Lhado Lam, Thimphu, Bhutan; 3 Boston College, 140 Commonwealth Avenue, Chestnut Hill, MA 02467 USA; 4 Khesar Gyalpo University of Medical Sciences of Bhutan, Menkhang Lam, Thimphu, Bhutan

**Keywords:** Community health worker, Bhutan, Village health worker, Hospital admissions

## Abstract

**Background:**

Village health workers (VHWs) in Bhutan play an all-encompassing role in supporting the health of their communities. Recent reports from the Bhutan Ministry of Health have indicated a sharp reduction in the number of working VHWs. As such, our work attempts to estimate the cost saved and the number of averted hospital admissions onto the Bhutanese healthcare system and the individuals who are served by these health workers.

**Methods:**

We utilized a dataset from the Bhutan Ministry of Health which encompassed over 95% of all reported disease cases within the nation. We examined the impact that VHWs have on hospital admission rates for eight diseases of interest by using multiple multivariate logistic regression models. Our model allowed us to estimate the potential disease cases averted when the average number of VHWs per health center is increased by one unit. Lastly, we utilized the 2011 “A Costing of Healthcare Services in Bhutan” to estimate the cost saved attributed to VHWs.

**Results:**

An average one unit increase of VHWs per health center is associated with a decrease in hospital and clinic admission for diarrhea, dysentery, wound care, depression/anxiety, dental caries, and skin infection, while a non-significant increase was observed for scabies and conjunctivitis. These findings translate to 4604 outpatient visits averted, with $28,637 saved, and 78 inpatient visits averted, with $10,711 saved. These values sum to a total of 4682 yearly averted admissions at health centers, with a total cost savings of $39,348 yearly. Additionally, we estimated a yearly savings of $13,348 in transportation costs and a total of $20,960 saved in wages to the community members that VHWs serve.

**Conclusions:**

VHWs serve as a source of cost-savings for the Kingdom of Bhutan and also act as an economic buffer for more vulnerable communities. The cost-savings associated with these health workers is likely to become more pertinent as the nation begins to develop and healthcare costs increase. It is imperative that proper action be taken to retain these health workers as every VHW who leaves the program increases healthcare costs onto the Bhutanese government.

## Background

The Kingdom of Bhutan is a small land-locked country situated in between the Indo-China Himalayan border. According to the World Health Organization (WHO), Bhutan spends just 3.6% of its gross domestic product (GDP) on health, but has managed to make significant strides in improving the overall health of its population in recent decades [1]. Immunization coverage in Bhutan is maintained over 95%, while neonatal tetanus, leprosy, iodine deficiency disorders, and malaria have been largely eliminated. Bhutan’s health promotion efforts are largely overseen by the Ministry of Health, while health services are delivered via a three-tiered system: Basic Health Units I and II (BHUs) at the primary level, district hospitals at the secondary level, and the national and referral hospitals at the tertiary level. However, government financing constraints have placed significant limitations on the nation’s ability to further invest in its health [1]. It is among these reasons that VHWs, who are not financially compensated, act as a cost-free mechanism to better the health of their communities [2]. By bridging cultural gaps that other healthcare professionals may bear, VHWs provide companionship, use their common identity, and provide perspective to deliver educational and health-related resources [3]. Research has indicated the benefits of VHWs, in terms of both health outcomes and cost-effectiveness, in a variety of economic settings [2]. However, to our knowledge, little to no research has delineated the quantitative effect of VHWs on hospital/clinic admission rates in developing regions. A primary reason for this may be that, unlike Bhutan, the majority of nations utilizing VHWs do not have this program integrated as a foundational all-encompassing pillar of their healthcare system; making it increasingly difficult to analyze the effects of VHWs onto an entire health system. Additionally, many cost-saving studies on VHWs have often focused on including only the saved cost incurred onto a greater healthcare system [4]. Our findings consider this metric, while also investigating some of the more unseen factors that may be attributed to VHWs, such as saved wages and transportation costs. This study aims to quantitatively model the impact that VHWs have on health center admissions as well as extrapolate the various cost savings achieved by such health workers. Due to the unique healthcare structure of Bhutan, the country remains a powerful model for better understanding the impact that VHWs have on a national scale as well as on the communities that these health workers serve.

## Methods

### Data collection

With the support of the Bhutan Ministry of Health, we collected hospital and clinic admissions data for the following illness/maladies: conjunctivitis, scabies, diarrhea, dysentery, wound care (bites and stings, contact with heat, work related injury, and other injury), depression/anxiety, dental caries, and skin infections. The classification of these diseases was diagnosed by a Bhutanese healthcare provider and the data were subsequently aggregated by the Ministry of Health into a central database. More specifically, a disease health admission was classified as such if a health worker recorded that either the primary cause of admission was due to that disease or if the patient presented with the disease-like symptoms. This data set encompassed over 95% of all the reported disease cases in the country of Bhutan; over 300,000 health center admission cases for our eight maladies of interest.

### Statistical model

**Fig. 1:**
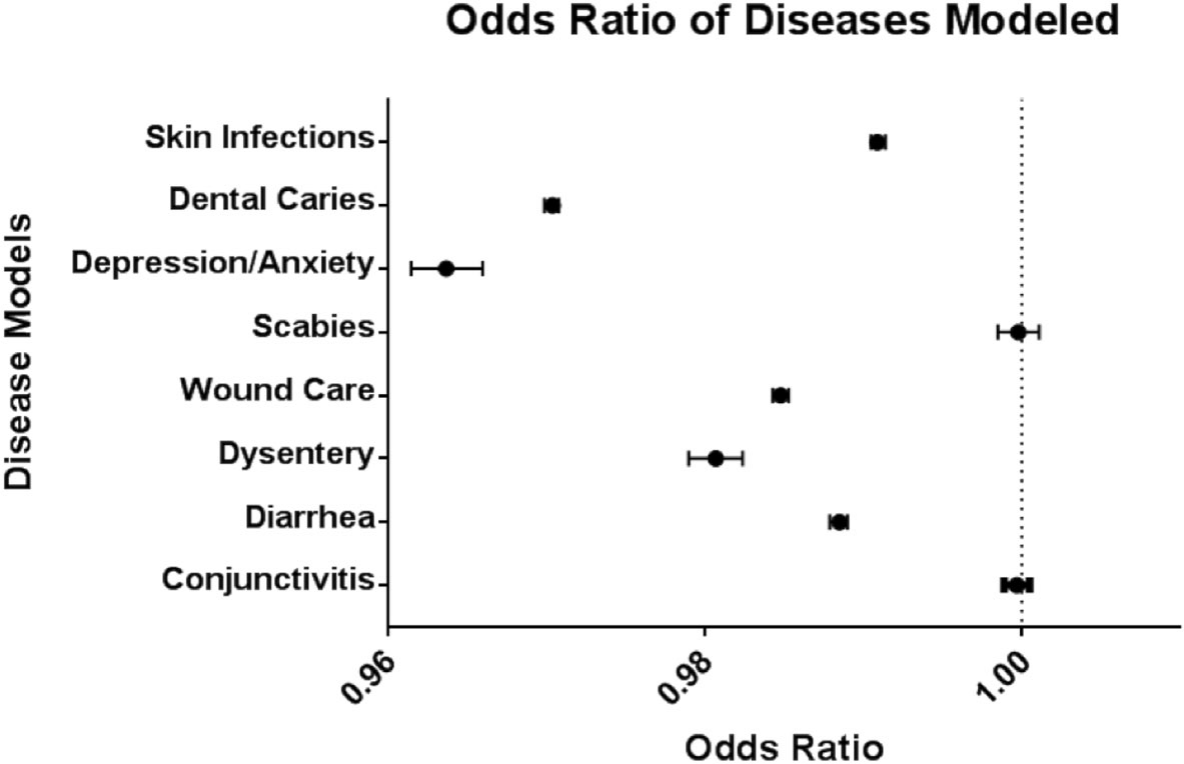
The OR estimates, along with the 97.5% CI, for the average impact of a one unit increase in VHWs per health center on health center admissions rates for the eight diseases examined within Bhutan

Using the latest data on health center admissions, we calculated the number of potential disease cases averted by increasing the average number of VHWs per health centers by one unit; which for our model reflected adding 42 VHWs to the nation of Bhutan. To quantify the impact on our dependent variable, admission rates for diseases of interest, we ran multiple multivariate logistic regression models using grouped data for our eight diseases, which VHWs are tasked with handling - either directly or indirectly. Utilizing VHWs as the independent variable, we were able to evaluate the impact that these health workers have on reducing disease admissions (see Table 1 and Fig. 1). We controlled for regional discrepancies, population age and size, number of VHWs per community, and health disparities per district (using reported number of illnesses per month as a proxy variable). We report odds ratios (OR) as the odds that a health admission for the disease of interest would occur if there were a one-unit increase in VHWs per health center. To assess the fit of our models, we will report McFadden’s Pseudo-R^2^ and perform a Deviance goodness-of-fit to determine the overall fit of our regressions. R version 3.6.1 was utilized in conducting all of the statistical analysis for this study.

### Cost-saving calculations

Using the 2011 “A Costing of Healthcare Services in Bhutan,” released by the Bhutanese Policy and Planning Division [5], and our results from the multivariate logistic regression model, we calculated the economic impact of increasing the average number of VHWs per health center by one unit. Our group stratified the total number of admissions for the eight-specific illness/maladies we modeled by the three types of Bhutanese healthcare facilities (Basic Health Units, District Hospitals, and Referral Hospitals) – which each have starkly different costs for admission. We then further stratified for the number of admissions and associated costs that were outpatient vs. inpatient. Using the average unit cost of admission in each healthcare facility type, we were able to calculate the average estimated cost-saving associated with a one unit increase in VHWs per health center (see Tables 2 and 3). More specifically, the OR for each model was multiplied by the annual number of admissions for each disease; this revealed the number of averted cases. The number of averted cases was then multiplied by the average unit cost at each facility type, producing the cost saved in Bhutanese ngultrum- ngultrums were then converted to U. S dollars. The average unit cost is reported by the Bhutan Ministry of Health as the cost associated for admitting an individual to a health center. Additionally, we collected data from approximately 200 Basic Health Units with regards to the average travel time a community member has to spend to reach their clinic. To calculate the saved costs relating to transportation, we multiplied the number of averted cases by the average transportation costs, $4.21, related to reaching a health center in Bhutan; the average transportation costs was determined independently by the Bhutan Ministry of Health [5]. To find the potential cost-savings from lost wages we multiplied the number of averted cases within the inpatient category by the average inpatient length of stay, 5.1 days, within a Bhutanese health center as reported by the Ministry of Health [5]. This value was then multiplied by the average daily Bhutanese salary to produce the estimated cost-savings related to wages.

### Ethical considerations

The appropriate data was retrieved from a central database developed by the Bhutan Ministry of Health. Such data did not contain any personal identifiers and had been previously collected for purposes external to our investigation.

## Results

### Discussion

Our estimates show a yearly cost savings of roughly a thousand dollar and over 100 averted cases for every VHW added to the country of Bhutan, with three-quarters of cost saving and the grand majority of averted cases arising from the outpatient’s department. These figures reveal that VHWs serve as a cost-saving mechanism onto the Bhutanese healthcare system and reduce the number of health center admissions for the diseases modeled. Additionally, we found over $800 saved yearly in wages and transportation for every additional VHW added to Bhutan; in which close to 40% arose from transportation cost and the rest from lost wages. These are notable components to consider as the work VHWs provide proves to be a substantial factor in avoiding lost wages and incurred cost in low-resource communities. Prior studies have extensively outlined the cost-effectiveness and cost-saving potential associated with community health workers (CHWs) in a variety of settings [6, 7]. A 2015 literature review of CHWs in India, Pakistan, Bangladesh, and Nepal shows that these health workers reduced costs when compared to standard of healthcare for both patients and providers with regards to a variety of health services provided (i.e. neonatal care, maternal education, mental health) [8]. While our research does support a clear economic argument for investing in CHWs, the cost-saving figures produced may seem like a small portion of the total Bhutanese healthcare budget. The primary reason for this is that the Kingdom of Bhutan holds a surprisingly low cost of admittance to a health center; as low as $2.32 per admittance. As the country of Bhutan continues to develop and healthcare costs continue to grow, the cost-saving associated with VHWs will very likely become more evident. Additionally, exporting our model to neighboring nations, such as Nepal or India, which hold significantly higher average cost of health center admittance, $6.58 and $18.90 respectively, would very likely see a much greater cost-savings associated with VHWs [9, 10].

**Table 1:** Summary of our disease models from the multivariate logistic regression after controlling for the defined parameters within Bhutan

Disease Models	OR for Village Health Worker (VHW) Count	97·5% Confidence Interval	***p***-value	Pseudo-R^**2**^ (McFadden’s)	Deviance (G^**2**^)
Conjunctivitis	0.9997	(0.9989, 1.0005)	ns	0.65	16095***
Diarrhea	0.9885	(0.9879, 0.9890)	*P* < .001	0.74	324756***
Dysentery	0.9807	(0.9790, 0.9824)	P < .001	0.63	4505***
Wound Care	0.9848	(0.9843, 0.9853)	P < .001	0.78	49345***
Scabies	0.9998	(0.9985, 1.0011)	ns	0.64	6639***
Depression/Anxiety	0.9637	(0.9615, 0.9660)	P < .001	0.65	4050***
Dental Caries	0.9704	(0.9699, 0.9708)	P < .001	0.54	50493***
Skin Infections	0.9909	(0.9905, 0.9914)	P < .001	0.72	60077***

P-Value for Deviance Goodness-of-Fit Test:

* *p* < 0.05, ** *p* < 0.01, *** *p* < 0.001

Table 1 shows that after adjusting for other covariates, our logit models indicate that an average one unit increase of VHWs per health center is associated with a decrease in hospital/clinic admission for diarrhea (OR 0.9885, 97.5%CI 0.9879, 0.9890), dysentery (OR 0.9807, 97.5%CI 0.9790, 0.9824), wound care (OR 0.9848, 97.5%CI 0.9843, 0.9853), depression/anxiety (OR 0.9637, 97.5%CI 0.9615, 0.9660), dental caries (OR 0.9704, 97.5%CI 0.9699, 0.9708), and skin infection (OR 0.9909, 97.5%CI 0.9905, 0.9914), while a non-significant increase was observed for scabies (OR 0.9998, 97.5%CI 0.9985, 10,011) and conjunctivitis (OR 0.9997, 97.5%CI 0.9989, 1.0005). For model fit, Table 1 details key fit descriptors by reporting the *McFadden’s* Pseudo-R^2^ and deviance statistics for each of the models. The deviance statistics were all significant, thereby indicating that our models were superior to their null (intercept-only) counterparts. Lastly, the high *McFadden’s* Pseudo-R^2^ values also indicates that the model is well fit. The level of signifcane for each of the deviance tests is reflected by * p < 0.05, ** p < 0.01, *** p < 0.001

**Table 2:** Number of averted admissions, stratified by inpatient visit (IPV) and outpatient visit (OPV), per one-unit average increase in VHWs per health center. Table also shows the associated cost saved for each stratified category

Disease Models	No. Outpatient Visit Averted (Cost Saved in United States Dollar)	No. Inpatient Visit Averted (Cost Saved in United States Dollar)	Total No. Cases Averted (Cost Saved in United States Dollar)
Diarrhea	456 ($1646)	26 ($2984)	482 ($4630)
Dysentery	107 ($386)	8 ($967)	115 ($1353)
Wound Care	926 ($20,020)		926 ($20,020)
Depression/Anxiety	109 ($717)	30 ($5038)	139 ($5755)
Dental Caries	2232 ($3056)		2232 ($3056)
Skin Infections	774 ($2812)	14 ($1722)	788 ($4534)
Total	4604 ($28,637)	78 ($10,711)	4682 ($39,348)

Table 2 displays the reported OR estimates for each of the disease models, we estimate 4604 OPV averted, with $28,637 saved, and 78 IPV averted, with $10,711 saved. These values sum to a total of 4682 averted admissions at health centers, with a total cost savings of $39,348. Based on our model of 42 Bhutanese health centers, an addition of one VHW to the nation of Bhutan would generate a total cost saving of $937 and avert 111 cases; total cost saved and total number of averted cases ($39,348 and 4682) divided by the number of health centers modeled (42)

**Table 3:** Total savings in transportation and wages associated with a one-unit average increase in VHWs per health center within Bhutan

Disease Models	Transportation Cost Saved in United States Dollar	Outpatient Visit Wages Saved in United States Dollar	Inpatient Visit Wages Saved in United States Dollar	Total Wages Saved in United States Dollar	Total Savings in United States Dollar
Diarrhea	$2035	$2067	$1011	$3078	$5113
Dysentery	$481	$710	$303	$1013	$1494
Wound Care	$3903	$5996	··	$5996	$9899
Depression/Anxiety	$581	$501	$1133	$1634	$2215
Skin Infections	$3323	$5074	$555	$5629	$8952
Dental Caries	$3025	$3610	··	$3610	$6635
Total	$13,348	$17,958	$3002	$20,960	$34,308

Table 3 displays the reported OR estimates for each of the disease models, we estimate $13,348 saved in transportation costs. We found a total of $20,960 saved in wages ($17,958 savings in wages from OPV, $3002 savings in wages from IPV). These values amount to a total of $34,308 saved for every one unit increase in VHWs per health center or $817 saved for every additional VHW added to Bhutan; total cost saved ($34,308) divided by the number of health centers modeled (42)

The model utilized took into account eight maladies which VHWs manage in some form. For all but two disease models, scabies and conjunctivitis, there was a significant reduction in health center admission rates. In particular for two maladies examined, depression/anxiety and dental caries, there was the most significant drop in the odds of visiting a health center. With regards to dental caries, the decrease in likelihood of visiting a health center likely arose from VHWs efforts to promote dental hygiene. Globally, CHWs are being used to educate patients on dental hygiene and oral health by connecting individuals to healthcare resources and teaching basic dental hygiene. A 2009 study conducted in southeastern Brazil found significant changes in perception regarding oral health, an increase in tooth brushing and flossing, and an increase in the self-assessment of oral hygiene efficacy due to CHWs [11]. This is especially valuable as our research supports the notion that having CHWs promote dental health can serve as an effective tool in decreasing health center admission rates for tooth decay as well as encourage proper dental hygiene practices.

As for the disease model of depression, Bhutanese VHWs are not formally trained to manage individuals suffering from depression or anxiety. However, for many rural and impoverished communities the only available source of emotional and psychological support may come from these health workers. There has been accumulating evidence over the years supporting the continued usage of training for CHWs to address mental health disparities and to improve care for underserved communities. The World Health Organization’s director of Mental Health and Substance Abuse stated that not only are community mental health services more accessible to people with mental disabilities, but are also more effective compared to treatments typically received at mental hospitals [12]. There have also been numerous studies supporting this notion, such as in São Paulo, Brazil where researchers found that symptoms of depression significantly improved amongst patients who were visited by CHWs [13]. Similarly, a 2018 systematic review, which highlighted Pakistan, Burma, Cambodia, and Nepal also found improved mental health for individuals who had support from CHWs [14]. It is hence, not by chance that out of the eight-disease modeled, the data related to depression/anxiety had the lowest OR. These findings, along with past research, reinforce the notion that CHWs can be utilized as an effective cost-saving measure to reduce health disparities with regards to mental health.

The following disease models predominantly reflect the fact that VHWs are able to rapidly provide simple remedies medications, and treatments to their communities: diarrhea, dysentery, wound care, skin infections. It is possible that a portion of the decrease in admission rates for these diseases is due to VHWs emphasizing preventive measures such as safe working habits and proper food-storage practices. However, it is more likely that this decrease arises from community members, particularly those who reside far from health centers, opting to acquire medications from their local VHW rather than from their nearest clinic. Intuitively, studies have shown that communities far from health centers especially benefit from utilizing CHWs as a rapid and reliable source for basic medications and other health-related resources [15, 16]. Additionally, it is worth mentioning that the category of wound care saw the most significant cost-savings and number of averted cases associated with the model; encompassing 50% of the total cost saved and 20% of the total number of averted cases. This is due to the fact that VHWs are able to provide rapid first-aid and wound sterilization for many non-serious injuries; a common service that VHWs provide and one which is well advertised to Bhutanese communities.

Lastly, it is important to note that there is significant benefit to communities acquiring medications and wound care rapidly as the likelihood of disease progression and disease morbidity are significantly hampered when treatment is made readily available. More specifically, having access to medications which treat or reduce symptoms of diarrhea, dysentery, or skin infections as well as having VHWs present to sterilize wounds, lead to both decreased risk of progression/infection and discomforts experienced by patients. This is particularly important as a key barrier to treatment is often access to health centers due to lengthy travel time or structural barriers in commuting [17]. Hence, there is not only an economic value of saved wages and incurred cost onto communities, but also a health component in the form of decreased morbidity due to VHWs rapidly providing medications and health resources that may otherwise be difficult to acquire.

### Strengths and limitation

The unique structure of this research allowed us to quantitatively measure the impact that VHWs have on expanding access to health resources and their associated economic impact by using health center admissions as a proxy. This was predominately made possible because Bhutan has a nation-wide VHWs program and affords its citizens free universal healthcare; thus, allowing this research to relate health admissions with VHW’s impact. However, our investigations were only able to model eight diseases. It is very likely that VHWs may also decrease health center admission rates for other maladies, most notably diseases such as the common cold and the flu. It is for this reason, that we believe that the figures provided represent a minimum of cost savings and number of averted cases. Additionally, the majority of cost figures were derived from the averages of each specific health center category. Due to this, it is possible that the average cost of admittance at a specific health center is not reflective of the actual cost of admittance for the specific disease modeled. Likewise, transportation costs and wages were modeled using the national average cost associated with transportation to and from health centers and the median Bhutanese salary, respectively. With regards to the portion of our research which developed the cost saved, we were unable to calculate the possible cost saved from VHWs influence on preventing and containing disease outbreaks; a task which they are assigned with monitoring. Lastly, the data regarding dental caries were reported in total number of caries instead of total number of individuals. In order to calculate the cost saved with regards to transportation cost and wages, we assumed that data from the capital city of Thimphu was representative of the total Bhutanese population with regards to average number of carries per person.

## Conclusions

CHWs in a variety of economic and social settings have shown to increase access to health resources in a cost-effective manner. As support for the usage of CHWs expands, developing nations face increasing strain with the retention of these health workers. In Bhutan alone, there has been a ~ 20% decrease in VHWs since 1992 [1]. This is evidently alarming as each VHW lost produces the reciprocal of our findings - an increase of $937 added to the Bhutanese healthcare system and an increase in 111 health center admittances per year. Similarly, the lost wages and transportation costs also incur proportionally to our findings. As such, the economic argument for investing into Bhutan’s CHWs is evident as our research has delineated the program as a cost saving initiative. Independent of the healthcare savings associated with VHWs, a substantial effort should still be undertaken to ensure that the Bhutanese VHWs program is revitalized, as it expands vital health resources to key communities, serves as an economic buffer for vulnerable groups, and aids to reduce disease morbidity.

## Data Availability

All relevant data remains with the Bhutan Ministry of Health. Administrative permissions are necessary to obtain the respective data through the Bhutan Ministry of Health. Appropriate requests can be made at: info@health.gov.bt.

## Abbreviations

CHWs: Community Health Workers
VHWs: Village Health Workers
WHO: World Health Organization
